# Notes and News

**Published:** 1890-07-12

**Authors:** 


### Notes and News.

Collected and Communicated.

The annual report of Great Yarmouth Hospital is in every way satisfactory. The committee were rather anxious about funds for carrying on the work of their enlarged buildings, but are happily able to state that their income has exceeded their expenditure by £98. Six new members have been elected to the committee. Both house surgeon and matron are thanked for the thorough manner in which they have carried out their duties during the past year.

It is to be regretted that Hospital Saturday at Newcastle ended in a fracas, during which ex-Mayor Whittingham assaulted one of his fellow workers. The culprit has been fined and bound over to keep the peace, but the whole proceedings in the police court showed what at the best can be called an undignified meeting. Of course such a case has caused much sensation in Newcastle, and the Fund cannot but be injured by the conduct of its members.

We are glad to see a movement is being made to establish a cottage hospital in Spain, in the Cadiz district. An appeal for £200 for this object is signed by William Moore, of the Missionary Training College, and Joseph Viliesed, of Jerez. It is a pity the appeal is prefaced with stories of the ill-treatment of Protestant patients in the Catholic hospitals which now exist, and in which, by Royal decree, a room is always set apart for non-Catholics. Some of these stories are too disgraceful to be readily believed; for instance, last year a German Protestant sailor fell ill, and his Consul sent him to the hospital, paying for him. Every day, when the Consul's clerk came to inquire for the patient, the Sisters would entreat him to speak to him of the Virgin, and, as he refused, they neglected the poor fellow, until he became so ill that he was unable to turn himself in his bed, and then these heartless Sisters hardly ever came near him, nor even helped him to turn, which caused him to contract bed sores. Feeling that the bed-clothing was not sufficiently warm, he requested that his thick ulster should be thrown over the bed; this also was refused, and he died rather from want of care than from the disease.

Dr. Drysdale has written a long letter to the *Echo* on the subject of the effects of tobacco on French men of letters. Victor Hugo, Balzac, Dumas the elder, Heine, and Michelet never smoked; but then it seems that De Musset, Eugene Sue, and George Sand did, so that it is six of one and half a-dozen of the other. There are always two sides to a question, but perhaps it is as well enthusiasts can see but one, or we should have neither martyrs nor reformers. As Oscar Wilde says in his facile and flippant way, "It is well for our vanity that we slay the criminal, for if we suffered him to live he might show us what we had gained by his crime. It is well for his peace that the saint goes to his martyrdom: he is spared the sight of his harvest."

The same dealer in epigrams has a sentence which the Charity Organisation Society might like for a motto—"Charity creates a multitude of evils." And talking of this organisation, there is a capital story of one of its Australian devotees, Professor Morris, who, while inquiring into philanthropic by-ways in Boston, demanded of an authority who he considered had most benefited the poor. "Octavia Hill and St. Paul," was the prompt reply. Perhaps it was rather rough on St. Paul to put him second, but then he left out one word in his celebrated text on charity; he ought to have written—"Though I give all my goods to feed the poor and have not charity organisation, it profiteth me nothing."

There is a pretty tale in verse in this month's *Good Words*, supposed to be told by a house-surgeon who finds the hospital ward "a wonderful place for seeing the truth of His Word." It tells of an old farmer who comes to the infirmary with a crushed arm, and who explains to the house surgeon his family troubles, the greatest of which is that one boy "grew restless, and went away." It so happens that this boy—now a young man—is dying in the next ward worn out by his life of sin. The father sees him by chance :—

> And I saw, as he gazed, how the look grew intense and keen, and a sudden pain,

> A terrible conflict of anguish and doubt, seemed surging through heart and brain;

> Then the dying man, who had closed his eyes as if done with the life we live,

> Opened them gently, stretched out his arms, and murmured, "Father, forgive!"

>

> "Oh, David, my son!" from the pale lips rang the "exceeding bitter cry,"

> As he knelt by the bed, held the dying hands, and looked in the dying eye;

> And the sound of the wings of the angel of death seemed all of a sudden to cease,

> Till the angels of penitence, pardon, and love should whisper, "Depart in peace."

The opening of the New French Hospital last week was interesting because of the foreign flavour given to the proceedings by the amount of French spoken all around one. M. Waddington, the French Ambassador, performed the ceremony, and was presented with bouquet of flowers of the national colours. In the course of his speech, M. Waddington said he thought the necessity for a hospital had not been exaggerated, and he eulogized the efforts of the late M. Eugène Rimmel, who had taken an active part in its establishment, together with the officers, members of the committee, and subscribers, all of whom deserved their thanks. Having spoken in congratulatory terms of the union existing between French residents in London, he declared the institution open amid loud applause. Continuing, he said he had been charged by the President of the Republic to invest Sir William MacCormac, the chief surgeon, with the order of Officer of the Legion of Honour; Mr. T. Verity, the hon. architect, with Chevalier of the same order; and Mr. James Curtis, the hon. solicitor, and Mr. Ernest Ruffer, the hon. secretary, with the orders of Officer of the Academy. These gentlemen having received the orders and warrants at the hands of the ambassador, they each thanked the Republic for the recognition of their services, and the proceedings terminated.

The following vacancies are announced this week, the amount of the salary in each case being given after the name of the institution :—

**Resident Medical Officers.**

Carlisle Infirmary, Assistant House Surgeon—£40, board, etc.

East London Hospital for Children, House Surgeon—board, etc.

Bristol City Lunatic Asylum, Assistant Medical Officer—£150, board, etc.

Manchester Hospital for Consumption, Resident Medical Officer —£60, board, etc.

Stockport Infirmary, Assistant Medical Officer—£70, board, etc.

**Non-Resident Medical Officers.**

Victoria University, Leeds, Demonstrator of Anatomy and Demonstrator of Physiology—£150.

Parish of Fulham, Analyst—£75.

Vestry of St. Mary, Islington, Sanitary Superintendent—£200.

Infirmary for Consumption, Margaret Street, Physician, Three Visiting Physicians, and a Surgeon.

Eccles Medical Association, Assistant Medical Officer.

The following are the figures of last year's report of the Warwick Hospital Saturday Fund, made public at a meeting held on June 25th: Collected by sheets, £69 0s. 6½d. ; by boxes, £31 6s. 8¼d. ; by balance last year, £1 18s. 9d. ; total, £102 5s. 11¾d. The expenditure on management amounted to £12 5s. 6d., and the following grants had been made: Warneford Hospital, £55 ; Children's Ward, Warneford Hospital, £3 17s. 2½d. ; Warwick Cottage Hospital, £30 ; balance in hand, £1 3s. 4¼d. ; total, £102 5s. 11¾d. The Secretary also supplemented the balance-sheet by the statement that during the seven years in which collections had been made the total had amounted to £634 12s. 9½d., last year realising £100 7s. 2½d.

Sir Edwin Chadwick, the well-known advocate of all health subjects, died last Saturday, at the age of 88. He was a veteran attendant at the congresses of the Sanitary Science Association and of the British Association. He recognised to the full the importance of popularising the laws of health, and became one of the warmest friends and supporters of the National Health Society, which, under the chairmanship of his friend, Mr. Ernest Hart, has done so much to enlist the help of the people themselves—both men and women—in the cause of public health progress. In his last years he became president of the Association of Sanitary Inspectors, and, throughout, his sympathy and his help were always at the command of the humblest as well as of the highest workers in the cause which he had so much at heart, and which he did so much to advance. He had been created C.B. at an early period in his career, and he was lately promoted to the rank of K.C.B., in recognition of the value of his lifelong labours.

The informal opening of All Saints' Convalescent Home for Children has taken place. The premises are not yet complete, but one wing is finished, and into this the children have been moved. Members of the All Saints' community gathered in force, and the Rev. Father Maturin officiated at a dedicatory service. The front elevation of the building, when complete, will be striking and handsome. The total length will reach some 240 ft., with a depth in the main part of 56 ft. The building is of red brick, with blue Staffordshire bands and red roof tiling, with some stone pointing and capitals, to give relief. Two chief rooms are refectories, measuring 35 ft. by 24 ft., one for each sex. The lavatories upon every floor are elegantly fitted up, and fully answer all sanitary requirements. On the ground floor, entry is obtained to the babies' dormitories. All the sleeping apartments have a pitch of about 17 ft., and some of the larger measure 25 ft. by 24 ft. Those which are furnished present every appearance of comfort. As the hospital is dedicated to All Saints, so every room therein is dedicated to some particular saint, whose name appears above the exterior of the doorway. The total accommodation will give about 130 beds. Exclusive of the cost of the site and the furnishing, the outlay is estimated to reach about £12,000. The work, so far as it has been carried out, reflects high credit on Mr. A. Mardon Mowbray, the architect.

The annual meeting of the Fellows of the Royal College of Surgeons took place on July 3rd in the College in Lincoln's Inn Fields. There were three vacancies to be filled on the Council. The two candidates in favour of reform, who were also the nominees of the Association of Fellows, were Mr. Walter Rivinston and Mr. Lawson Tait, of Birmingham. Besides these surgeons there were five other candidates for the three vacancies. In an hour's time the result of the poll was declared, and Mr. William Mitchell Banks, of Liverpool, Mr. Marcus Beck, and Mr. John Langton were declared duly elected members of the Council for five years. Mr. Lawson Tait came fourth with 25 votes behind the third on the list. Mr. Beck is surgeon to University College Hospital, and Mr. Langton to St. Bartholomew's Hospital.

On July 2nd the Hospital Reform Committee met at Birmingham to consider the answers received to the papers of questions sent out by them. These questions dealt with the number of patients, their cost, etc. ; but the question on which reform will probably be based, and the answers to which are therefore most valuable was: Question 8: "What means, if any, are adopted to prevent the admission of persons able to pay for the treatment their cases require?" And Question 9 was: "How many were rejected on this ground?" The following are the replies :—

*General Hospital.*—Tickets are given at subscriber's discretion, who are requested to ascertain the social and pecuniary circumstances of each case. Any case apparently unsuitable for gratuitous treatment would be investigated, and if found in a position to pay for private advice would be refused treatment. Number rejected: No record ; believed to be very few.

*Queen's.*—A system of registration, by which all patients, other than accidents and emergencies are required to answer certain questions to the complete satisfaction of the registrar. Number rejected, 113.

*Dispensary.*—We have no means of ascertaining the circumstances of the patients, but every ticket patient must be recommended by a subscriber, who signs a definite form as to the case being a deserving one. Number rejected very few, but no record.

*Eye Hospital.*—In cases of doubt the circumstances are enquired into by the secretary, and dealt with accordingly. Very few are rejected—it is believed not more than 25, but no record has been kept.

*Children's.*—The secretary makes enquiries from applicants, and notices are posted in the waiting hall warning improper persons against applying for relief. Number rejected, 33.

*Women's.*—Enquiries are made as to the means of out-patients, but the decision is left to the judgment of the surgeons. Rejected, about six.

*Ear and Throat.*—Special enquiry by registrar. Rejected, none.

*Orthopædic.*—None, other than the investigation by the medical officers of the day. Cases have been rejected, but we have no record of the number.

*Skin and Lock.*—Enquiry of each applicant as to circumstances. Rejected, 96.

*Dental Hospital.*—Patients are required to state their employment, and if any doubt exists as to their fitness they are asked to show cause why they cannot afford to pay a private practitioner. No statistics kept of rejections, but they are not a large number.

*Homœopathic.*—(a) In-patients are questioned by the house surgeon as to their means. (b) Out-patients—printed on governors' notes of recommendation are questions to be answered as to the means of the patient. Cannot ascertain to what extent governors decline to give dispensary notes if the patient is able to pay. If a note be once granted no further questions are asked.

*Lying-in Charity.*—Questioned by secretary. Rejected, none.

*Medical Mission.*—Where there is any doubt the patients are, as a rule, questioned, and if found able to pay asked to get a doctor. Rejected, not 20.

*West Bromwich.*—Weekly earnings of the patients, or the head of the family, is stated on the notes of recommendation. Rejected, none.

